# Protective Role of *Ziziphus lotus* Fruits and Leaves Extracts Towards BPA‐Associated Testis Toxicity

**DOI:** 10.1002/fsn3.71911

**Published:** 2026-05-28

**Authors:** Dhekra Grami, Soumaya Wahabi, Raja Jouini, Imen Hlel, Naoures Ochi, Slimen Selmi, Hichem Sebai, Luca De Toni

**Affiliations:** ^1^ Laboratory of Functional Physiology and Valorization of Bioresources‐Higher Institute of Biotechnology of Beja University of Jendouba Beja Tunisia; ^2^ Pathology Departement, Faculty of Medecine of Tunis Habib Thameur Hospital, Tunis EL Manar University Tunis Tunisia; ^3^ Department of Medicine University of Padova Padova Italy

**Keywords:** bisphenol A, male reproductive toxicity, oxidative stress, rats, *Ziziphus lotus* fruit, *Ziziphus lotus* leaves

## Abstract

*Ziziphus lotus* L. is used in folk medicine for various ailments. This study investigated the potential protective effects of aqueous extracts from the fruit (ZLFAE) and leaves (ZLLAE) of *Z. lotus* against testicular toxicity associated with Bisphenol A (BPA) exposure in a rat experimental model. ZLFAE and ZLLAE were characterized for the content of phytochemicals, showing high content in catechol, quercetin, and caffeic acid for ZLFAE associated with higher antioxidant capacity. Male Wistar rats were divided into six groups orally exposed to BPA (100 mg/kg), or BPA combined with ZLFAE (150 or 300 mg/kg) or ZLLAE (150 or 300 mg/kg) for 4 weeks. In control animals (CTRL), exposure to BPA and treatments with ZLFAE or ZLLAE were omitted. Animals were then sacrificed and assessed for testis histology, semen parameters, antioxidant parameters, and oxidative stress biomarkers. Exposure to BPA was associated with reduced testis and epididymis weights, reduced serum testosterone and semen parameters, with increased markers of lipid peroxidation, oxidized thiols, and impaired catalase (CAT), glutathione peroxidase (GPx), and superoxide dismutase (SOD) activity. Treatment with *Z. lotus* extracts, particularly ZLFAE 150 mg/kg, markedly mitigated these biochemical and structural alterations. These findings suggest that *Ziziphus lotus* L. fruits exert protective effects likely due to their phenolic compounds with antioxidant properties, offering a potential protective potential towards BPA‐associated testicular toxicity.

## Graphical abstract

Aqueous extracts of *Ziziphus lotus* fruits and leaves show protective effects towards testis toxicity associated with oral bisphenol A exposure in a rat model, through the likely involvement of antioxidant activity.

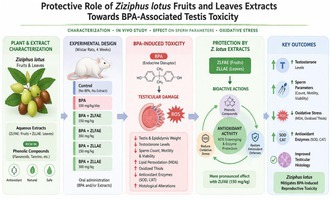

## 1 Introduction

Great attention is currently drawn by the harmful effects of chemicals substances able to interfere with hormonal balance, collectively known as endocrine‐disrupting chemicals (EDCs). Particularly, Bisphenol A (BPA) is recognized as an EDC (Metcalfe et al. 2022). BPA is widely used as an additive to several plastic materials such as polycarbonate and epoxy resin, resulting as almost ubiquitous in food containers, paper products, water pipes, toys, medical equipment, and electronic products (Manzoor et al. 2022). BPA spreading into the environment takes place at any stage of it the life‐cycle of the matrices containing it whilst its exposure is recognized to occur upon several routes including: ingestion, mother‐fetus transmission, inhalation and skin/mucosae/eye contact (Almeida et al. 2018). Exposure to BPA is suggested to have a role in clinical issues associated with obesity, diabetes, cardiovascular disease, thyroid dysfunction and cancer (Abd El Salam and El Sayed Selim 2019). Furthermore, available studies confirmed that BPA can act on the male reproductive system, ultimately affecting reproductive ability and contributing to couple infertility (De Toni et al. 2020; Mondal and Bandyopadhyay 2021; Selvaraju et al. 2021).

The molecular bases of BPA activity as EDC has been related to the partial agonism towards estrogen receptors *α* and *β*, associated with disruption of the central endocrine axis, reduction of testosterone levels and impairment of semen quality (Shamhari et al. 2021). Accordingly, BPA exposure in animal models has been associated with adverse effects in testis function, including decreased testis weight, decreased epididymal sperm concentration, decreased serum testosterone, and altered sperm morphology (Ryu et al. 2023). However, recent findings from animal models suggest a major role for BPA in triggering oxidative stress, resulting in lipid peroxidation, morphological changes and impaired sperm motility through the impairment of cell energy metabolism (Mukherjee et al. 2025).

*Ziziphus lotus* L. is popularly known as “Sedra,” belongs to the angiosperm *Rhamnaceae* family, including about 135–170 *Ziziphus* spp., and owns edible, orange‐yellow fruits called “Nbeg” (Messaad and Belhadj 2025). In Africa, *Z. lotus* is widely distributed in Mediterranean region, like Algeria, Morocco, Tunisia, and Libya (Bencheikh et al. 2023). Recent studies have reported that *Z. lotus* is rich in flavonoids, essential oils, tannins, phytosterols, triterpenoid, vitamin C, glycosides, saponins, and alkaloids, which are considered the major accountants for its favorable health effects (Almasri et al. 2025). In fact, in traditional medicine, the different parts of *Z. lotus* have been used as a popular remedy for several purposes, including anti‐bacterial, anti‐viral, anti‐diabetes, anti‐fever and anti‐diarrhea activity, as well as lenitive for genital‐urinary discomfort (Alla et al. 2025; Marmouzi et al. 2019). *Z. lotus* leaf extracts are currently used to ameliorate eye diseases, ulcers, bronchitis, wounds, and skin disorders, whilst fruit and bark extracts have demonstrated notable antimicrobial properties (Bakhtaoui et al. 2014; Bekkar et al. 2022; Bencheikh et al. 2023; El Yakoubi et al. 2024; Ghazi‐Yaker et al. 2024; Imran et al. 2023; Zazouli et al. 2022). Importantly, most of the biological activity of *Z. lotus* is suggested to be related to high content in antioxidant compounds (Messaad and Belhadj 2025).

While the best approach to preventing BPA‐associated clinical risk remains the reduction of exposure levels, possible approaches to address BPA‐related toxicity are based on its role in impairing the cell redox system (De Toni et al. 2020). Consequently, the use of natural sources of antioxidants has been widely evaluated, and our group recently demonstrated that primary outcomes associated with BPA exposure in animal models are significantly improved by treatment with antioxidant‐rich plant extracts (Grami et al. 2020). In this study, we aimed to investigate the potential protective role of two aqueous extracts from *Z. lotus* , obtained respectively from leaves (ZLLAE) and fruits (ZLFAE), in a rat model of BPA‐induced testis damage.

## 2 Materials and Methods

### 2.1 Chemicals

Bisphenol A (2,2‐Di (4‐hydroxyphenyl) propane; 97% purity), trichloroacetic acid (TCA), acetylcholine iodide, S‐butyrylcholine, butylhydroxytoluene (BHT), potassium hydroxide, ethanol, bovine serum albumin (BSA), acetylthiocholine iodide, 5.5′‐dithiobis‐(2‐nitrobenzoic acid) (DTNB), Triton X‐100, Eosin Y alcoholic solution, and Roswell Park Memorial Institute culture medium (RPMI) were purchased from Sigma‐Aldrich Chemie GmbH (Germany). Corn oil was purchased from a local commercial market.

### 2.2 Sample Collection and Aqueous Extract Preparation

*Ziziphus lotus* L. leaves and fruits were collected during August 2023 from the area of Beja, in the north area of Tunisia, and identified by the botanist Chokri Hafsi (Center of Biotechnogy of Borj Cédria—Higher Institute of Biotechnolgy of Beja (ISBB), University of Jendouba, Tunisia). Fresh leaves and fruit were dried in an incubator at 40°C for 72 h and then ground into powder using an electric grinder. The plant powder was subsequently dispersed in distilled water and incubated at room temperature for 24 h under magnetic stirring. The homogenate was filtered through a 0.5 mm mesh size filter, the filtrate lyophilized, aliquoted and stored at −80°C until use. The extraction yield was calculated as 100 × (weight of dried extract (g)/weight of dried plant material). All extractions were performed in triplicate to ensure reproducibility and reliability of the results.

### 2.3 Profile of Phenolic Compounds by HPLC‐ESI‐MS

100 mg of each extract were dissolved in 100 mL of 10% methanol, filtered by 0.2 μm pore size nylon membrane filters (Millipore‐Sigma Aldrich), and 1 mL was transferred into LC–MS glass vials. A reverse‐phase column (Pursuit XRs ULTRA 2.8, C18, 100 × 2 mm, Agilent Technologies, UK) was used to perform HPLC analysis. 20 μL of the prepared samples were injected at a column temperature set at 30°C. Mobile phases consisted of 0.1% formic acid in water (A) and 0.1% formic acid in methanol (B). A gradient elution was used at a flow rate of 1 mL/min. Mobile phases gradient consisted of an initial composition of 100% solvent A, with a gradient of 100% solvent B over 20 min, held at 100% solvent B for 5 min and 100% solvent A for 25 min. The drying gas flow rate was 1 mL/min at 320°C. MS was operated in the positive ion mode in a mass range of 100–2000 *m/z*. High‐resolution mass spectral data were obtained on a Thermo Instruments ESI‐MS system (LTQ XL/LTQ Orbitrap Discovery, UK) connected to a Thermo Instruments HPLC system (Accela PDA Detector, Accela PDA Autosampler, and Accela Pump).

### 2.4 Phytochemical Screening

The total phenolic content in the aqueous extract of ZLLAE was quantified using the Folin–Ciocalteu colorimetric method (Lamuela‐Raventós 2017). This method is based on the reduction of the Folin–Ciocalteu reagent by phenolic compounds under alkaline conditions, leading to the formation of a blue‐colored complex determinable by spectrophotometry at 765 nm. Brief, 100 μL of extract samples were mixed with 1/10 (v/v) diluted Folin–Ciocalteu reagent and 7.5% Na_2_CO_3_ solution. After incubation for 30 min at room temperature, the absorbance was then recorded. Gallic acid was used as the reference and results were expressed as milligrams of gallic acid equivalents (mg GAE/g extract).

The total flavonoid content was determined using the aluminum chloride colorimetric method (Chang et al. 2020). This method is based on the formation of a stable complex between aluminum chloride and the hydroxyl groups of flavonoids, producing a yellow coloration whose intensity is proportional to the flavonoid concentration. Briefly, 500 μL of extract was mixed with 1.5 mL of methanol, 0.1 mL of 10% aluminum chloride, 0.1 mL of 1 M potassium acetate, and 2.8 mL of distilled water. After incubation at room temperature for 30 min, the absorbance of the reaction mixture was measured at 415 nm with a UV spectrophotometer. The amount of 10% aluminum chloride was substituted by the same amount of distilled water in the blank. Total flavonoid content was expressed as catechin equivalents (mg CE/g extract).

The total tannin content was determined using the Folin–Ciocalteu colorimetric method, with tannic acid used as standard (Makkar 2003). This method is based on the oxidation/reduction reaction between phenolic hydroxyl groups of tannins and the Folin–Ciocalteu reagent under alkaline conditions, resulting in the formation of a blue‐colored complex whose intensity is proportional to the tannin concentration.

Briefly, 0.5 mL of the extract solution was mixed with 0.5 mL of 50% Folin–Ciocalteu reagent and allowed to incubate for 2–5 min. Then, 1.0 mL of 20% sodium carbonate solution was added. After incubation for 10 min at room temperature, the mixture was centrifuged for 5 min at 1000×*g*, and the absorbance of the supernatant was measured at 730 nm. The total tannin content was expressed as milligrams of tannic acid equivalents per gram of extract (mg TAE/g).

The total antioxidant capacity (TAC), assessed by the 2,2‐diphenyl‐1‐picrylhydrazyl (DPPH) radical scavenging activity, and the ferric‐reducing power (FRP) were assessed as previously described (Ben Barka et al. 2017; Wahabi et al. 2023; Yıldırım et al. 2000).

### 2.5 Animal Treatments

Animal procedures were approved by the Biomedical Ethics Committee of the Pasteur Institute of Tunis (approval no. JORT472001; April 15, 2024). All experiments were conducted in accordance with the National Institutes of Health (NIH) guidelines for the care and use of laboratory animals and the ARRIVE guidelines for reporting animal research (Percie du Sert et al. 2020).

Male Wistar rats were purchased from the Central Society of Pharmaceutical Industries of Tunisia (SIPHAT, Ben Arous, Tunisia). The study was performed according to the guidelines of the local Ethics Committee of Tunis University for the care and use of laboratory animals, in compliance with NIH recommendations (Couto 2011). Acquired animals were allowed to acclimatize for 2 weeks before any experimental procedures as per standard facility practice (Grami et al. 2020).

A preliminary evaluation of the acute toxicity of *Ziziphus lotus* was performed. Oral doses of ZLLAE or ZLFAE of respectively 500, 1600 and 3200 mg/kg were administered to groups of 6 Wistar rats per dose and extract. Animals were then closely monitored every 30 min over an initial period of 4‐h and followed by intermittent observations for an additional 8 h. Mortality was recorded up‐to 4 weeks post‐administration. In addition to the survival rate, animals were examined for major signs of toxicity, including alterations in motor coordination, righting reflex, and respiratory function.

In long‐term treatment study, 42 male Wistar rats, 8 weeks old with body weight ranging from 200 to 250 g, were purchased from SIPHAT (Ben Arous, Tunisia). Animals were housed in groups of six per cage in separate metallic cages under standard laboratory conditions (temperature 24°C ± 2°C, relative humidity 55% ± 5%, and a 12 h light/12 h dark cycle). They were fed with a standard pellet diet (Society of Badr Utique, Bizerte, Tunisia) containing 67% carbohydrates, 10% fat, and 23% protein (total energy: 3.6 kcal/g), with water provided ad libitum.

**FIGURE 1:**
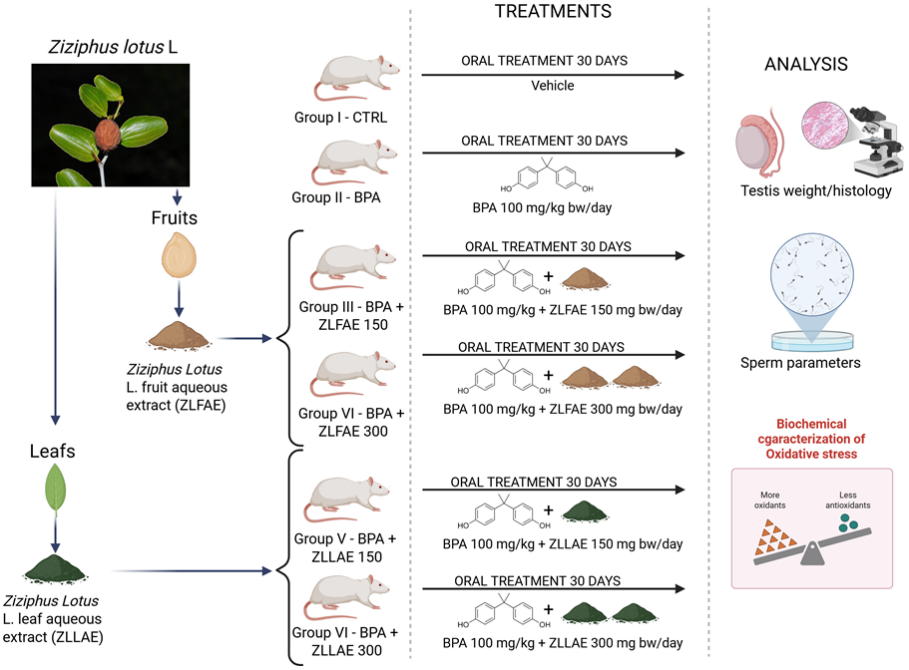
Representative scheme of the experimental design of the study aimed at evaluating the possible protective role of the aqueous extracts from *Ziziphus lotus* L., obtained respectively from leaves (ZLLAE) and fruits (ZLFAE), in a rat model of bisphenol A (BPA)‐induced testis damage. BW, body weight.

The experimental treatment involved 6 groups of animals, randomly assigned to each group in order to minimize selection bias. Experimental groups were defined as follows (Figure 1) :

- Group I (CTRL): rats treated with 0.4 mL/kg/day of tocopherol‐stripped corn oil in an equivalent volume to that given to the different experimental batches.
- Group II (BPA): rats treated with Bisphenol A at a dose of 100 mg/kg dissolved in tocopherol‐stripped corn oil.
- Group III (ZLFAE‐150): rats treated with *Ziziphus lotus* L. fruit aqueous extract at a dose of 150 mg/kg in association with BPA 100 mg/kg.
- Group IV (ZLFAE‐300): rats treated with *Ziziphus lotus* L. fruit aqueous extract at a dose of 300 mg/kg in association with BPA 100 mg/kg.
- Group V (ZLLAE‐150): rats treated with *Ziziphus lotus* L. leaves aqueous extract at a dose of 150 mg/kg in association BPA 100 mg/kg.
- Group VI (ZLLAE‐300): rats treated with *Ziziphus lotus* L. leaves aqueous extract at a dose of 300 mg/kg in association with BPA 100 mg/kg.

For the multiple comparison among 6 experimental groups, with a statistical power *β* of 0.8, a significance level *α* of 0.05, an effect size from previous studies of at least 0.6 units of standard deviation and an estimated dropout rate of 10% due to death or suffering of the animal, the sample size of each group was set at *N* = 7 animals (Sample Size Calculator: Power Analysis Tool).

All animals were treated orally for 4 weeks, in agreement with previous data research showing that this exposure period associates with measurable effects on the hypothalamus‐pituitary‐gonads axis and testicular function (Grami et al. 2020; Karnam et al. 2015).

After 4 weeks of treatment, all animals were sacrificed by cervical dislocation and the blood samples were collected by intracardiac withdrawing in tubes. Animal sera were obtained by whole blood centrifugation and used for the estimation of luteinizing hormone (LH), follicle‐stimulating hormone (FSH), and total testosterone levels. Hormone levels were measured by commercially available ELISA kits (rat testosterone ELISA kit, Cat. No. E90243; rat LH ELISA kit, Cat. No. CK‐E90904; and rat FSH ELISA kit, Cat. No. CK‐30597) according to the manufacturer's instructions (Bio Pharm Co., USA). Absorbance was read using a BioTek Synergy H1 microplate reader (BioTek Instruments, Winooski, VT, USA). Testis and the epididymis were immediately removed by dissection, weighed, and processed as described below. Then necessary, tissue samples were homogenized in phosphate‐buffered saline (pH 7.4). For biochemical parameter analysis, the homogenates were centrifuged at 3000×*g* for 10 min at 4°C using a Universal 320 R centrifuge (Andreas Hettich GmbH & Co., Germany). The resulting supernatants were stored at −80°C until use.

### 2.6 Animal Metrics and Reproductive Organs Assessment

Body weight of each rat was recorded at the time of the sacrifice. The absolute weights of the reproductive organs were recorded after carefully removing surrounding fat and blood vessels. Signs of reproductive organ toxicity were evaluated based on the presence of observable clinical symptoms.

Total sperm count, sperm motility, and morphology were evaluated from the right cauda epididymis, whilst the left testis and epididymis were used for biochemical assessment. Isolated epididymis was excised and minced in 1 mL of RPMI with 1000 UI penicillin/mL and 1000 μg of streptomycin/mL to obtain sperm suspension (Grami et al. 2020).

Cell count of cauda‐epididymis spermatozoa was performed as described elsewhere and expressed as total sperm count per epididymis (Grami et al. 2020). Briefly, ten microliters of sperm‐suspensions were rapidly layered onto a warmed microscope slide. Motility patterns were evaluated using the ATS20 Computer‐Aided Semen Analysis (CASA, JCD, Gauville, France) and distinguished into progressive motile, non‐progressive motile, and non‐motile spermatozoa, and reported as percentage of total sperm count (Bjorndahl 2002). The Eosin stain method was used to determine sperm viability. The staining procedure involved the use of 10 μL of freshly collected semen combined with 20 μL of eosin‐PBS solution. Viable sperms appeared as unstained whilst dead sperms showed purple to red staining. The dye exclusion was analyzed in 100 spermatozoa and results reported as the percentage of dead sperm cells (Tardif et al. 1999). Sperm suspensions were smeared on a glass slide, dried at room temperature, and stained with 1% eosin solution to evaluate sperm morphology according to Seed's method (Seed et al. 1996). One hundred spermatozoa obtained from different fields in each slide were analyzed and classified for measures of observed irregularities (head, tail, and tail‐head). Non‐typical morphology of sperm cells was reported as percentage (Filler 1993).

In order to minimize errors, a technical replicate on three separate preparations was assessed for each sample and the resulting mean value was used for subsequent analysis.

Testicular sperm production was estimated according to the procedure of Narayana et al. (2002) with slight modifications. Briefly, epididymal and testicular tissues were homogenized with 0.05% (v/v) Triton ×100/saline solution. Homogenates were then diluted with 1.5 mL of saline solution, and spermatozoa and spermatids were counted in three technical replicates. Mean data were reported as the number of mature sperm cells or spermatids per gram of organ, epididymis, or testis.

### 2.7 Biochemical Tissue Assessment

Protein concentration in the epididymis and testis supernatants was performed by Hartree technique (Hartree 1972) with few modifications to the Lowry method, using bovine serum albumin (BSA) as reference protein.

Lipid peroxidation in testis and epididymis supernatants was determined using the double‐heating method. Briefly, after protein precipitation with trichloroacetic acid, malondialdehyde (MDA), a marker of lipid peroxidation, was quantified by the reaction with thio‐barbituric acid (TBA) under acidic and high‐temperature conditions to form a pink chromogen (MDA–TBA complex), which was then quantified spectrophotometrically at 532 nm (Draper and Hadley 1990).

The quantification of total thiols content in testis and epididymis tissues was performed according to the method of Ellman (1959). Samples were mixed with 800 μL of phosphate buffer 0.25 M, pH 8.2, and 100 μL of EDTA 20 mM. The mixture was vortexed and the background absorbance (A1) was measured at 412 nm. Subsequently, 100 μL of 10 mM 5,5′‐dithiobis‐(2‐nitrobenzoic acid) (DTNB) were added to the reaction mixture, let incubate at 37°C for 15 min. and then a new absorbance reading (A2) was performed. The concentration of thiol groups was calculated as A2‐A1 (13.6 × 10^3^ mol/L × cm) as previously described (Ellman 1959). The results were expressed in nmol of thiol groups/mg proteins.

Superoxide Dismutase (SOD) activity was measured using the Misra and Fridovich method (Misra and Fridovich 1972). Briefly, epididymis or testis homogenates were mixed with 2 mL of a solution containing 20 μL of epinephrine 5 mg/mL, 10 μL of bovine catalase 0.4 U/μL and 62.5 mM of sodium carbonate/bicarbonate buffer pH 10.2. Absorbance variations were measured at 480 nm.

Catalase (CAT) activity was determined according to the method of Aebi (1984). The reaction mixture contained 33 mM hydrogen peroxide (H_2_O_2_) in 50 mM phosphate buffer (pH 7.0). The decay of H_2_O_2_ concentration was monitored at 240 nm and CAT activity was calculated using an extinction coefficient of 40 nmol/(L × cm) for hydrogen peroxide.

### 2.8 Epididymis and Testis Histology

For histological examination, specimens of testis or epididymis were fixed in 10% neutral buffered formalin solution and embedded in paraffin. Subsequently, 5 μm sections were laid on microscope slides, deparaffinized, hydrated, and stained with hematoxylin–eosin. The final preparations were microphotographed using an Olympus BH2 microscope fitted with photographic attachment (Olympus C35 AD4), a camera (Olympus C40 AB‐4), and an automatic light exposure unit (Olympus PM CS5P).

### 2.9 Statistical Analysis

Statistical analysis was performed with GraphPad Prism 10.6.1 (GraphPad Software, Boston, MA, USA). Data were expressed as mean ± standard deviation (SD). Statistical differences among groups were analyzed using one‐way ANOVA with post hoc Tukey's correction for multiple comparisons. Differences were considered statistically significant for values of *p* < 0.05.

## 3 Results

### 3.1 Characterization of *Ziziphus lotus* L. Leaves and Fruits Aqueous Extracts

**FIGURE 2:**
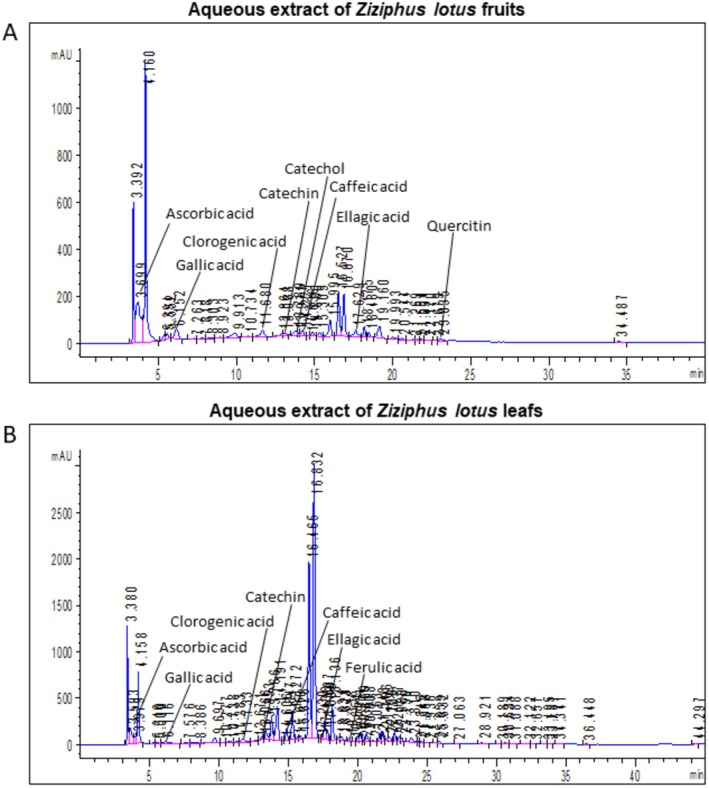
Representative phytochemical profiles of the aqueous extracts from *Ziziphus lotus* L. fruits (A) and leaves (B), obtained by liquid chromatography‐mass spectrometry (LC–MS). Indicated phytochemicals were identified by retention time and high‐resolution mass spectral data.

The mean extraction yields for ZLFAE and ZLLAE among three independent extraction lots were respectively: 6.8% ± 2.1% and 17.9% ± 4.3%. Aqueous extracts of *Ziziphus lotus* L. leaves and fruits were characterized by the HPLC‐ESI‐MS profile (Figure 2A,B and Table 1). Compounds such as ascorbic acid, gallic acid, chlorogenic acid, catechin, caffeic acid, and ellagic acid were identified in both extracts, although caffeic acid was highly represented in ZLFAE. On the other hand, catechol and quercetin were essentially found in ZLFAE, whilst ferulic acid was essentially detected in ZLLAE.

**TABLE 1:** Characterization of phytochemical profile of *Ziziphus lotus* fruits (ZLFAE) and leafs (ZLLAE) aqueous extracts by high‐performance liquid chromatography‐mass spectrometry.

	ZLFAE	ZLLAE
Ascorbic acid	2.290	3.990
gallic acid	0.634	0.659
Chlorogenic acid	0.685	0.510
Catechin	0.109	0.115
Catechol	2.429	n.d.
Caffeic acid	4.548	0.287
Ellagic acid	1.834	1.750
Ferulic acid	n.d.	0.685
Quercetin	0.218	n.d.

*Note:* Data are reported as μg/g of dry extract.

Abbreviation: n.d., not determinable.

ZLFAE and ZLLAE were also evaluated for the total content of polyphenols, flavonoids, and tannins, for which gallic acid, catechin, and tannic acid were respectively used as reference compounds. The antioxidant activity was also assessed, in comparison to ascorbic acid as a reference compound. Results are summarized in Table 2. In particular, ZLLAE showed a significantly higher content of total phenolics and flavonoids compared to ZLFAE but a lower content of condensed tannins. As a result, the total antioxidant capacity of both ZLFAE and ZLLAE was higher than that of ascorbic acid, but only ZLFAE had a higher DPPH index compared to the reference compound.

**TABLE 2:** Characterization of aqueous extracts of *Ziziphus lotus* L. fruits (ZLFAE) and leaves (ZLLAE) for antioxidant content and antioxidant activity.

	ZLFAE	ZLLAE	Ascorbic acid	*p*
Total phenolics (mg GAE/g DW)	26.7 ± 3.9	36.4 ± 1.1	//	**0.0143**
Flavonoids (mg CE/g DW)	14.1 ± 1.4	45.3 ± 3.0	//	**< 0.001**
Condensed tannins (mg CE/g DW)	3.0 ± 0.5	1.0 ± 0.2	//	**0.0023**
TAC (μg/mL)	52.1 ± 4.6	46.9 ± 1.7	6.9 ± 0.1	**0.0374** ^**a**^ **0.0026** ^**b**^ 0.1406^c^
DPPH IC_50_ (μg/mL)	74.9 ± 0.2	92.3 ± 0.3	71.7 ± 0.2	**0.0001** ^**a**^ 0.8163^b^ 0.8183^c^
FRAP IC_50_ (μg/mL)	7.1 ± 0.2	23.1 ± 0.1	3.2 ± 0.1	0.5635^a^ 0.8120^b^ 0.8137^c^

*Note: p* value at analysis of variance with post hoc Bonferroni correction, are reported. Comparisons: ^a^ZLFAE vs. Ascorbic acid; ^b^ZLLAE vs. Ascorbic acid; ^c^ZLFAE vs. ZLLAE. Significant *p* values are reported in bold. Data are reported as the mean value ± standard deviation (SD) of 3 independent experiments.

Abbreviations: CE, catechin equivalent; DPPH, 2,2‐diphenyl‐1‐picrylhydrazyl scavenging activity; DW, dry weight; FRAP, ferric‐reducing antioxidant power; GAE, gallic acid equivalent; TAC, Total antioxidant capacity.

### 3.2 Effect of BPA Exposure and *Ziziphus lotus* L. Treatment on Animal Anthropometrics and Reproductive Organs

A preliminary evaluation of the acute toxicity of *Ziziphus lotus* leaves and fruits aqueous extracts was performed. Neither deaths nor signs of acute toxicity outcomes, including alterations in motor coordination, righting reflex, and respiratory function, were recorded upon treatment with 500 to 3200 mg/kg single dose of either ZLFAE or ZLLAE during the following 24‐h monitoring (data not shown). On these bases, the estimated LD50 value is higher than the highest dose of 3200 mg/kg used in the current experimental conditions. This finding is consistent with previous studies reporting that *Ziziphus lotus* extracts exhibit low toxicity, with an estimated hemimaximal lethal dose (LD_50_) exceeding 5000 mg/kg body weight (Bekkar et al. 2022). Accordingly, daily dosages of 150 and 300 mg/kg bw were considered as safe in the subsequent long‐term treatment study as previously described (Bencheikh et al. 2023).

**FIGURE 3:**
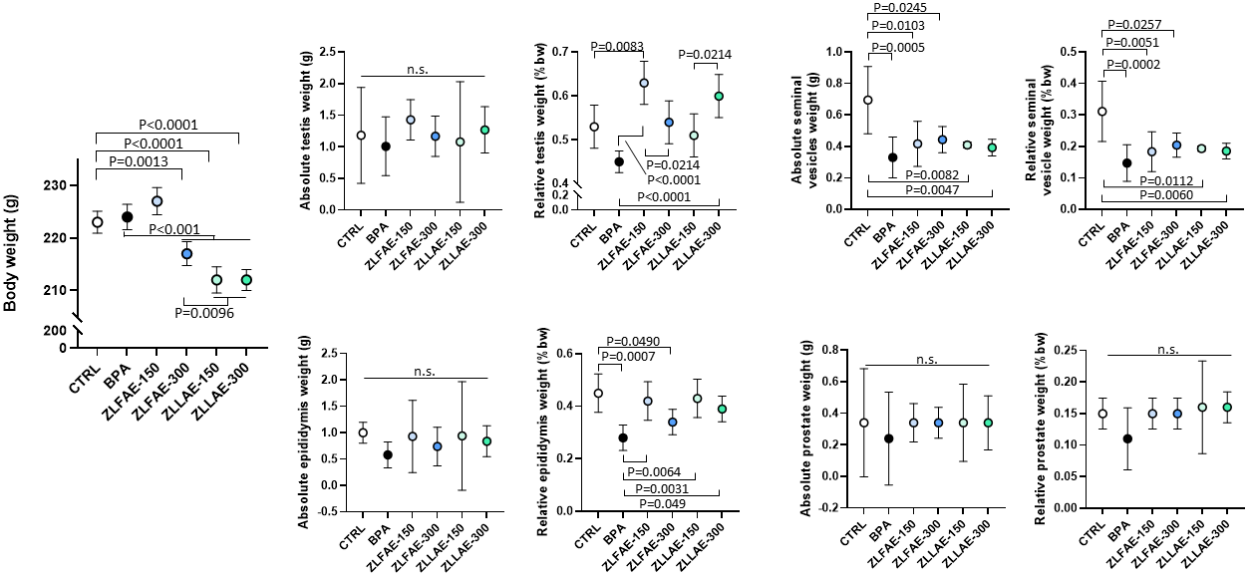
Analysis of body weight and reproductive organ weight, both absolute and relative to body weights, in Wistar rats orally exposed for 4 weeks to 100 mg/kg Bisphenol A (BPA), BPA 100 mg/kg with *Ziziphus lotus* L. fruit aqueous extract (ZLFAE) at a dose of 150 or 300 mg/kg or BPA 100 mg/kg with *Ziziphus lotus* L. leaves aqueous extract (ZLLAE) at a dose of 150 or 300 mg/kg. In the control group (CTRL), any exposure/treatment was omitted. In each experimental group, animals were used. Data in plots are reported as the mean value ± standard deviation (SD, error bars) of *N* = 7 independent assessments, one for each animal in the treatment group. Significance: *p* value among experimental conditions at analysis of variance with post hoc Tukey's correction is reported; ns, non significant.

In long‐term treatment evaluation, animals were orally exposed to 100 mg/mL BPA or exposed to BPA and treated with ZLFAE or ZLLAE, either at a daily dose of 150 or 300 mg/kg, for 4 weeks. Results on the absolute and relative weight, as % of the body weight (bw), of animals and reproductive organs, including epididymis, testis, prostate, and seminal vesicles at the end of treatment are reported in Figure 3. Compared to CTRL animals, exposure to BPA had no effect on the body weight. On the other hand, treatment with ZLFAE, in association with BPA, had differential effects on this parameter since only the dose of 300 mg/kg was associated with a slight but significant reduction of the body weight compared to CTRL and the exposure to the sole BPA. Differently, both doses of ZLLAE resulted in a comparable and significant reduction of the body weight compared to both CTRL and BPA. Although there were some significant inter‐treatment differences, ZLFAE 300 mg/kg, ZLLAE 150 mg/kg and ZLLAE 150 mg/kg showed no variation compared to CTRL.

The evaluation of testis, epididymis, and prostate absolute weights showed no significant differences among experimental conditions. However, the assessment of the relative weight showed some peculiar pattern according to the specific organ. In fact, exposure to BPA had no significant effect on the relative weight of this organ compared to CTRL, whilst the treatment with ZLFAE 150 mg/kg was associated with a significant increase of this parameter compared to both CTRL and BPA.

Data on the relative epididymis weight showed more homogeneity. Whilst the exposure to BPA was associated with a significant reduction of this parameter, the association with ZLFAE 150 mg/kg, ZLLAE 150 or 300 mg/kg showed a significant and consistent recovery.

Data on seminal vesicles showed a peculiar pattern since exposure to BPA was associated with a significant reduction both in absolute weight and in relative weight. Concomitant treatment with ZLFAE or ZLLAE at either dose showed no significant recovery compared to BPA. Finally, prostate weight was largely unaffected by the different experimental conditions.

### 3.3 Effect of BPA Exposure and *Ziziphus lotus* L. on Testis Histology, Testis Function and Reproductive Hormones

The effect on testis histology, testis function and reproductive hormones associated with long‐term exposure to 100 mg/mL BPA, or exposure to BPA and treatment with ZLFAE or ZLLAE, either at a daily dose of 150 or 300 mg/kg, was assessed.

**FIGURE 4:**
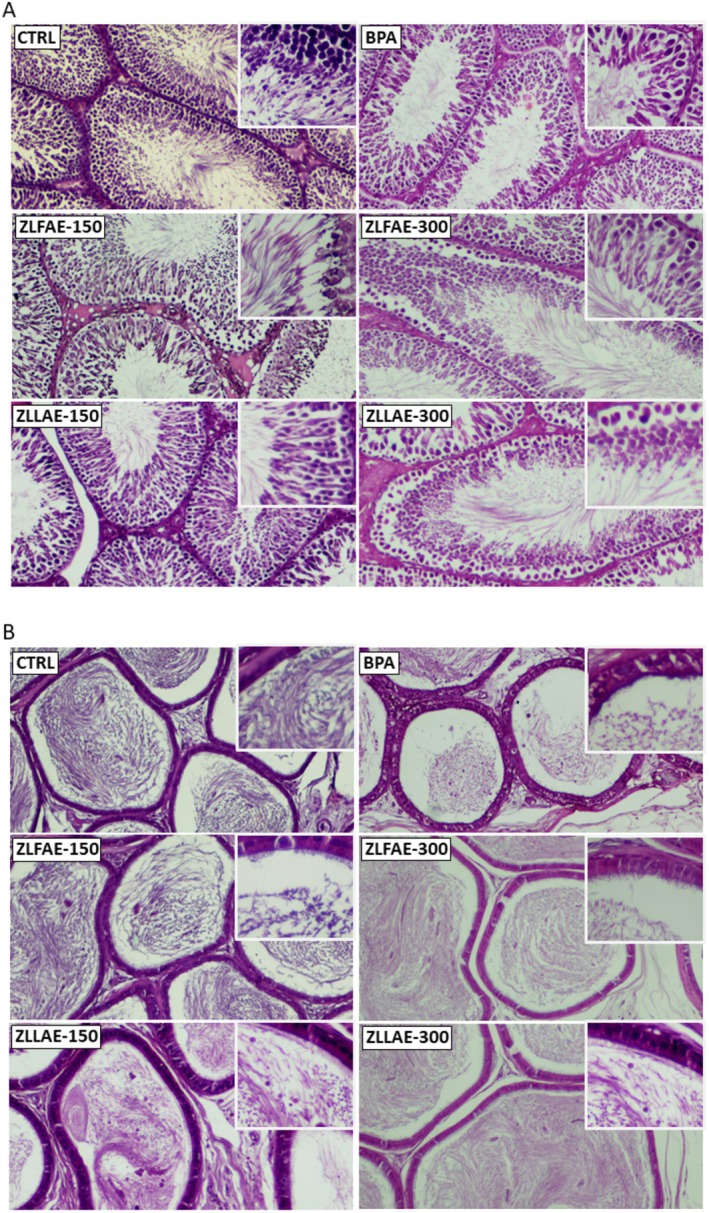
Representative images of testis and epididymis histology, evaluated by hematoxylin–eosin staining, in Wistar rats orally exposed for 4 weeks to 100 mg/kg Bisphenol A (BPA), BPA 100 mg/kg with *Ziziphus lotus* L. fruit aqueous extract (ZLFAE) at a dose of 150 or 300 mg/kg or BPA 100 mg/kg with *Ziziphus lotus* L. leaves aqueous extract (ZLLAE) at a dose of 150 or 300 mg/kg. In the control group (CTRL), any exposure/treatment was omitted. Pictures were captured at 10× magnification. Inserts in each picture were captured at 100× magnification to detail the representation of the germ line cells in the testis or epithelium thickness in the epididymis.

Representative images of the histological evaluation of testis and epididymis specimens in animal groups (Figure 4A,B) show that CTRL animals display a typical histo‐architecture characterized by regularly arranged seminiferous tubules, surrounded by connective tissue and interstitial cells. Tubules were lined with a stratified germinal epithelium composed of Sertoli and germ line cells. These cells were observed at various stages of spermatogenesis: spermatogonia with dark, small nuclei located along the basement membrane, spermatocytes with larger and more prominent nuclei, and spermatids in different stages of development, including elongated spermatids and spermatozoa. Animals exposed to BPA exhibited marked degeneration and disruption of the seminiferous epithelium, with substantial loss of the germ‐cell population, particularly spermatocytes and spermatids, and evidence of cellular degeneration or necrosis. The lumens of many seminiferous tubules contained disorganized and exfoliated cells.

Animals exposed to BPA and treated with ZLFAE 150 mg/kg showed a partial preservation of testicular histo‐architecture. A comparable histological pattern was observed also in animals exposed to BPA and treated with ZLFAE 300 mg/kg, ZLLAE 150 mg/kg, or ZLLAE 300 mg/kg.

In control animals, the epididymal epithelium was normal with a lumen filled with spermatozoa. BPA exposure caused mild epithelial disorganization and reduced sperm density. Treatment with 150 mg/kg ZLFAE or ZLLAE at either dose was associated with preserved epididymal structure and luminal sperm content.

**FIGURE 5:**
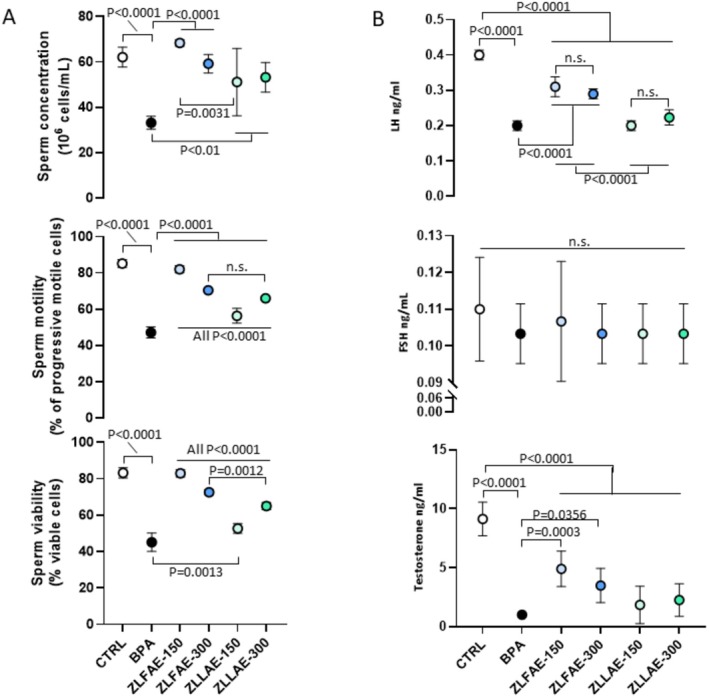
Analysis of semen parameters and reproductive hormones, including luteinizing hormone (LH) and follicle stimulating hormone (FSH), in Wistar rats orally exposed for 4 weeks to 100 mg/kg Bisphenol A (BPA), BPA 100 mg/kg with *Ziziphus lotus* L. fruit aqueous extract (ZLFAE) at a dose of 150 or 300 mg/kg or BPA 100 mg/kg with *Ziziphus lotus* L. leaves aqueous extract (ZLLAE) at a dose of 150 or 300 mg/kg. In the control group (CTRL), any exposure/treatment was omitted. Data in plots are reported as the mean value ± standard deviation (SD, error bars) of *N* = 7 independent assessments, one for each animal in the treatment group. Significance: *p* value among experimental conditions at analysis of variance with post hoc Tukey's correction is reported; ns, non significant.

The effect on testis function and reproductive hormones is reported in Figure 5A,B. The quantitative evaluation of sperm parameters (Figure 5A) showed a consistent, as well as expected, massive reduction of the total sperm count, progressive motility, and viability upon exposure to BPA, compared to CTRL. The treatment with *Ziziphus lotus* L. was associated with a significant recovery of sperm parameters compared to BPA, although some differential pattern could be observed. In fact, treatment with ZLFAE 150 or 300 mg/kg was associated with a nearly complete recovery of all sperm parameters. On the other hand, treatment with ZLLAE 150 mg/kg was associated with the lowest increase of all sperm parameters compared to BPA.

The serum pattern of reproductive hormones was also assessed (Figure 5B). A strict consistency was observed between serum levels of LH and T with semen parameters since, compared to CTRL, exposure to BPA was associated with a significant reduction of both hormone levels. In general, treatment with *Ziziphus lotus* L. extract in BPA‐exposed animals was associated with a significant increase of LH levels, with a greater extent of recovery associated with ZLFAE. However, only ZLFAE were associated with a significant increase of serum T compared to BPA. However, no treatment was associated with the restoration of T values to those of controls.

Serum FSH showed to be unaffected by any experimental condition.

### 3.4 Effect of BPA Exposure and *Ziziphus lotus* L. Treatment on Biochemical Parameters of Testis and Epididymis

**FIGURE 6:**
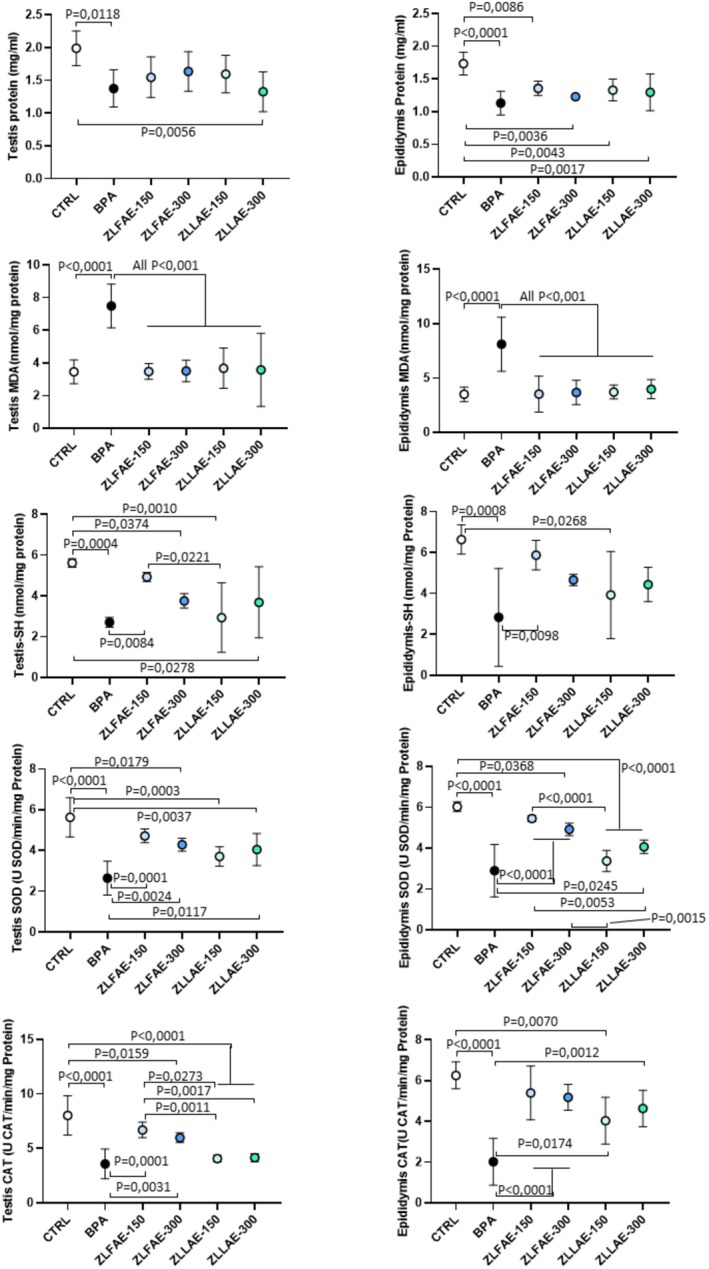
Analysis of testis and epididymis biochemical parameters, including total protein content, malondialdehyde (MDA) value, thiol content (−SH), superoxide dismutase (SOD) activity and catalase activity (CAT), in Wistar rats orally exposed for 4 weeks to 100 mg/kg Bisphenol A (BPA), BPA 100 mg/kg with *Ziziphus lotus* L. fruit aqueous extract (ZLFAE) at a dose of 150 or 300 mg/kg or BPA 100 mg/kg with *Ziziphus lotus* L. leaves aqueous extract (ZLLAE) at a dose of 150 or 300 mg/kg. In control group (CTRL) any exposure/treatment was omitted. Data in plots are reported as the mean value ± standard deviation (SD, error bars) of *N* = 7 independent assessments, one for each animal in the treatment group. Significance: *p* value among experimental conditions at analysis of variance with post hoc Tuckey's correction, are reported; ns, non significant.

The effect on biochemical parameters associated with long‐term exposure to 100 mg/mL BPA, or exposure to BPA and treatment with ZLFAE or ZLLAE, either at a daily dose of 150 or 300 mg/kg, on testis and epididymis is reported in Figure 6.

In general, the total protein content of both testis and epididymis was reduced upon exposure to BPA. The total protein content of the testis of animals exposed to BPA and treated with ZLFAE or ZLLAE 150 mg/kg was not different compared to CTRL, although it was slightly reduced. Differently, the treatment with ZLLAE 300 mg/kg showed a significant reduction compared to CTRL. On the other hand, the total protein content of the epididymis was significantly reduced compared to CTRL upon exposure to BPA, and neither treatment with ZLFAE nor treatment with ZLLAE, at any of the tested dosages, was associated with a significant recovery of this parameter.

Results of the MDA test showed a significant increase in lipid peroxidation levels in both the testis and epididymis from animals exposed to BPA compared to CTRL, whilst both ZLFAE and ZLLAE treatment, independently of the dosage, were associated with significant recovery to control levels.

The evaluation thiol content, SOD and CAT activities of testis and epididymis showed a similar pattern according to the experimental condition. In fact, BPA exposure was associated with a significant reduction of all parameters in both tissues compared to CTRL. The concomitant treatment with ZLFAE, and particularly with ZLFAE 150 mg/kg, was associated with a significant and consistent recovery of all parameters in both tissues. On the other hand, only concomitant treatment with ZLLAE 300 mg/kg was associated with some significant recovery of SOD and CAT activities in the epididymis.

## 4 Discussion

In the present study we provide evidence that the treatment aqueous extracts of *Ziziphus lotus* L. fruits and leaves associate with a significant improvement of testis, semen and hormonal parameters in a recognized in vivo model of endocrine disruption represented by male rodents exposed to bisphenol A. In particular, the treatment with aqueous extract of *Ziziphus lotus* L. fruits at the dosage of 150 mg/kg showed the highest degree of recovery from the semen, hormonal and testis antioxidant machinery derangement associated with the BPA disruptive activity.

Our findings support a major role of BPA as a disruptor of the hypothalamic–pituitary–testicular axis since both LH and T are largely reduced by exposure to the pollutant. This associates with a significant derangement of androgen‐sensitive organs, such as the testis and seminal vesicles, together with massive impairment of the spermatogenic function (Hejmej et al. 2011). However, available data suggest that the reproductive outcomes associated with BPA exposure go beyond the mere effect of endocrine disruption. Tissue exposure to BPA has also been associated with the impairment of the cell redox balance, mitochondrial dysfunction, and oxidative stress (Grami et al. 2018; Meli et al. 2020). This is suggested to trigger the Fas/FasL signaling pathway, resulting in increased apoptosis of the germ line cells (Li et al. 2009). In line with this hypothesis, we found that exposure to BPA was associated with the impairment of all biochemical markers of oxidative stress, such as increased lipid peroxidation and severely reduced thiol content, superoxide dismutase, and catalase activity in both testis and epididymis tissues.

Antioxidants have been suggested as a nutritional based approach to overcome cell derangements associated with BPA‐related oxidative stress (Amjad et al. 2020). Several studies have investigated natural compounds as potential therapeutic sources of antioxidants for preventing and treating the reproductive toxicity associated with the exposure to endocrine disruptors such as BPA (Noh et al. 2020). Our phytochemical profiling of *Z. lotus* fruits and leaves depicts plant parts as sources of natural antioxidants, such as flavonoids, phenolic acids, and tannins. These data are consistent with those from other Moroccan studies identifying 22 phenolic compounds, such as gallic acid, pyrogallol, chlorogenic acid, catechin, rutin, and caffeic acid in *Z. lotus* extracts (Amjad et al. 2020; Bencheikh et al. 2019; Noh et al. 2020; Rached et al. 2019). However, some major differences in antioxidant composition were observed between ZLFAE and ZLLAE, the former featured by a higher total antioxidant capacity and the latter being richer in total phenolics and flavonoids, in agreement with data previously reported by Marmouzi et al. (2019).

This characteristic appears as non‐negligible, being associated with the effects observed in animals exposed to BPA and treated with ZLFAE and ZLLAE at different dose. In fact, whilst some improvement from BPA exposure was observed with both extracts, treatment with ZFLAE 150 mg/kg was associated with the highest degree of recovery of both semen, hormonal and biochemical parameters. Despite we provided no mechanistic investigation to explain this differential effect, it might be speculated that the differential effect on testis function might be related to the specific phytochemical composition of ZLFAE, featured by the higher content in catechol, caffeic acid and quercitin. Interestingly, previous data reported that caffeic acid was able to restore sperm derangements and hormonal imbalance in a rat model of senescence associated with D‐galactose exposure (Khoshdel et al. 2022). Similarly, the flavonoid quercitine improved germ cell apoptosis in adult rat exposed to estradiol‐3‐benzoate as an animal model of male testist derangement by estrogenization (Bharti et al. 2014). However, no data are currently available for catechol, prompting for the experimental assessment of the biological effects of this tannin derivative.

We acknowledge some drawbacks of the present study. First and foremost, it is a preclinical study with screening purposes aimed to identify some preliminary data on the effect of *Z. lotus* extracts on a model of testis derangement which was obtained by the exposure to a single dose of BPA. Indeed, this model does not reflect typical human exposure, rather resembling the upper limits of occupational exposure observed in epidemiological studies (Zhuang et al. 2015). Still, a daily oral dose of 100 mg/kg bw is widely used to assess toxicological issues in experimental murine models, particularly in regard to fertility and reproduction outcomes (Aja et al. 2023; Kamel et al. 2018; Mariem et al. 2025; Ogwoni et al. 2024; Zhuang et al. 2015). Accordingly, this dose regimen was adopted to obtain a clear status of endocrine and testis derangement on which to test the effect of plant extract. Nonetheless, the extrapolation to human health outcomes should be made with caution. In addition, a mechanistic proof supporting the differential effect of ZLFAE and ZLLAE is lacking. The qualitative histological, biochemical and hormonal evaluation of a recognized animal model represents a major strength supporting our results, though recognizing that the study would have benefitted from the histological scoring systems such as the Johnsen score. Further studies will properly address the specific role of *Z. lotus* extracts composition in the related biological effect.

## 5 Conclusions

In conclusion, our study suggests a promising potential for *Ziziphus lotus* aqueous extracts of seeds and leaves to mitigate the testis damage induced by bisphenol A. Differential improvement was observed between seeds and leaves, with greater effectiveness than the former plant part, possibly related to the higher content in caffeic acid and quercetin, previously shown to address testis derangements in experimental animal models. However, further studies are warranted to elucidate the precise molecular mechanisms involved and to explore its potential application in clinical or preventive settings.

## Author Contributions

**Soumaya Wahabi:** methodology, visualization. **Naoures Ochi:** methodology. **Imen Hlel:** methodology. **Dhekra Grami:** conceptualization, methodology, visualization, formal analysis, writing – original draft. **Hichem Sebai:** conceptualization, methodology, formal analysis, supervision. **Luca De Toni:** methodology, formal analysis, supervision. **Raja Jouini:** methodology. **Slimen Selmi:** methodology, visualization.

## Funding

The authors have nothing to report.

## Ethics Statement

All animal procedures were approved by the Biomedical Ethics Committee of the Pasteur Institute of Tunis (approval no. JORT472001; April 15, 2024) and were conducted in accordance with the National Institutes of Health (NIH) guidelines for the care and use of laboratory animals and the ARRIVE guidelines for reporting animal research.

## Conflicts of Interest

The authors declare no conflicts of interest.

## Data Availability

The data that support the findings of this study are available from the corresponding author upon reasonable request.
